# T staging esophageal tumors with x rays

**DOI:** 10.1364/OPTICA.501948

**Published:** 2024-04-19

**Authors:** T. Partridge, P. Wolfson, J. Jiang, L. Massimi, A. Astolfo, N. Djurabekova, S. Savvidis, C. J. Maughan Jones, C. K. Hagen, E. Millard, W. Shorrock, R. M. Waltham, I. G. Haig, D. Bate, K. M. A. Ho, H. Mc Bain, A. Wilson, A. Hogan, H. Delaney, A. Liyadipita, A. P. Levine, K. Dawas, B. Mohammadi, Y. A. Qureshi, M. D. Chouhan, S. A. Taylor, M. Mughal, P. R. T. Munro, M. Endrizzi, M. Novelli, L. B. Lovat, A. Olivo

**Affiliations:** 1 Department of Medical Physics and Biomedical Engineering, University College London, London WC1E 6BT, UK; 2 Division of Surgery and Interventional Science, UCL, London WC1E 6BT, UK; 3 Current address: Advanced Photon Source, Argonne National Laboratory, Lemont, Illinois 60439, USA; 4 Nikon X-Tek Systems Ltd., Tring, Herts HP23 4JX, UK; 5 Department of Computer Science, UCL, London WC1E 6BT, UK; 6 Department of Histopathology, UCL, London WC1E 6BT, UK; 7 Department of Upper Gastro-Intestinal Surgery, UCLH, London NW1 2BU, UK; 8 Center for Medical Imaging, Division of Medicine, UCL, London WC1E 6BT, UK; 9 Princess Alexandra Hospital Medical Imaging Department, Brisbane, Queensland, Australia; 10 University of Queensland Medical School, Saint Lucia, Queensland, Australia; 11 Research Department of Pathology, Cancer Institute, UCLH, London NW1 2BU, UK

## Abstract

With histopathology results typically taking several days, the ability to stage tumors during interventions could provide a step change in various cancer interventions. X-ray technology has advanced significantly in recent years with the introduction of phase-based imaging methods. These have been adapted for use in standard labs rather than specialized facilities such as synchrotrons, and approaches that enable fast 3D scans with conventional x-ray sources have been developed. This opens the possibility to produce 3D images with enhanced soft tissue contrast at a level of detail comparable to histopathology, in times sufficiently short to be compatible with use during surgical interventions. In this paper we discuss the application of one such approach to human esophagi obtained from esophagectomy interventions. We demonstrate that the image quality is sufficiently high to enable tumor T staging based on the x-ray datasets alone. Alongside detection of involved margins with potentially life-saving implications, staging tumors intra-operatively has the potential to change patient pathways, facilitating optimization of therapeutic interventions during the procedure itself. Besides a prospective intra-operative use, the availability of high-quality 3D images of entire esophageal tumors can support histopathological characterization, from enabling “right slice first time” approaches to understanding the histopathology in the full 3D context of the surrounding tumor environment.

## 1. INTRODUCTION

Recently there has been a significant interest in developing advanced, phase-based x-ray techniques that can support, and ideally replace, histopathology (“virtual histology” methods) thanks to their enhanced sensitivity to differences in soft tissues, using both synchrotron [1] and conventional sources [2]. The introduction of “single-shot” phase retrieval methods working with conventional sources [3] has allowed speeding up acquisitions to the extent where they become compatible with intra-operative use [4], while providing an image quality comparable to the histopathology standard [5]. This is part of a wider, global effort in the development of phase-based x-ray technology, which, following pioneering experiments based on crystal interferometers in the mid-1960s [6], saw a significant expansion in the mid-1990s, with propagation [7] and crystal-based [8] methods being developed both at synchrotrons and with laboratory micro-focal sources [9,10]. A significant step forward was obtained in the mid-2000s with the introduction of laboratory-based methods working with non-micro-focal sources based on grating-interferometry [11] or apertured masks [12], which were followed by a plethora of additional approaches utilizing, e.g., single grids [13], sandpaper [14,15], or other random phase objects [16] as modulators. These methods have been applied across the life and physical sciences with medicine often taking a predominant position [17]; notably, the *in vivo* stage has been reached in lung imaging [18] and, albeit with synchrotron radiation, mammography [19]; more recently, the possibility to integrate phase-based x-ray imaging on a clinical computed tomography (CT) gantry was also demonstrated [20].

Here we demonstrate the possibility to stage tumors on the basis of x-ray images alone, albeit limited to the T staging of excised esophagi. Tumors are staged according to the international TNM staging system where T is the degree of infiltration of the tumor into the primary organ, N represents involvement of adjacent lymph nodes, and M denotes the presence of distant metastases [21]. In the esophagus, T1 tumors involve only mucosa, whereas T4 tumors have infiltrated the entire esophageal wall into surrounding organs such as the diaphragm or aorta. Although this was not included in our study protocol, extending the method to N staging should be possible, as suggested by the detection of tumor infiltration in a lymph node that was accidentally included in one of the scans (see Supplement 1). The obtained image quality is sufficiently high to determine the degree of penetration of the tumor into the underlying tissue layers, and this is supported by direct comparison with conventional histopathology. In particular, two experienced radiologists were asked to blindly stage 14 images from tumor-bearing samples collected in the study, and we report the results matched to histopathology in all cases.

We note that, while histopathology is destructive, inherently 2D and only available post-operatively, the proposed approach is non-destructive, 3D, and could be made available during the surgical intervention itself, thus allowing the surgical team to adapt the intervention in real time. The benefit of a non-destructive technique in an intra-operative context is that it does not affect the resected organ’s ability to undergo all other stages of clinical evaluation (e.g., histopathology), and therefore it can provide additional information without affecting the clinical workflow.

We targeted esophageal tumors as this was identified as a “cancer of unmet need” by Cancer Research UK [22] and other institutions. Cancer of the esophagus is the seventh most common type of cancer worldwide [23], and its prevalence has continued to rise over the past 30 years [24]. Although five-year survival rates have improved from around 4% in 1970, they remain poor at 17% for patients diagnosed up to 2017 [24]. Early disease detection is associated with higher survival rates and better quality of life, particularly following the introduction of minimally invasive therapies, such as radiofrequency ablation (RFA) and endoscopic mucosal resection (EMR) [25].

Clinical staging of cancer is the most accurate reflection of prognosis and guides therapy. While this is typically done *in vivo* pre-operatively using imaging modalities (mostly CT but also including any combination of endoscopic ultrasound, positron emission tomography, and laparoscopy), inaccurate staging may also play a role in the relatively high incidence of tumor recurrence post attempted curative esophagectomy. Indeed, a large UK retrospective study found recurrence rates of 46.7% for both esophageal squamous cell carcinoma and esophageal adenocarcinoma [26]. It should also be noted that staging based on imaging modalities is prone to error; e.g., a meta-analysis of T2N0 staging showed an accuracy for T&N staging of just 19±4% , and T staging accuracy of 29±5% [27], even when imaging modalities were combined. In a recent large European study, 8.5% of patients underwent R1 esophageal resection, where R1 indicates the removal of macroscopic disease but microscopic margins positive for tumor. This was associated with significantly worse prognosis, reducing from 66 months to 24 months in N0 disease [28]. Correct intraoperative staging of the tumor would therefore permit further esophageal resection to happen, which should lead to significant prognostic benefits.

One reason why accurate local staging is problematic with conventional CT is because the contrast is generated by differences in attenuation between the tissue types. Soft tissues have limited attenuation differences, which can lead to poor image contrast [29]. As mentioned above, x-ray phase contrast imaging (XPCI), which relies on the phase changes that x rays undergo when traversing different tissue types, can solve this problem by providing much higher soft tissue contrast. Indeed, a previous example exists showing that XPCI can resolve the structure of esophageal layers where conventional CT struggles [30]; this, however, was conducted with synchrotron radiation, while here we focus on the use of conventional x-ray sources, as would be required by a prospective translation to clinical use.

Among the various approaches available to perform XPCI, we focus here on edge illumination (EI) [31], because, despite the use of optical elements to enable the detection of phase effects with relatively large focal spots, it is compatible with flyscans (i.e., continuous as opposed to step-and-shoot sample rotation). This has previously allowed the acquisition of full CT datasets in a few minutes in standard laboratory conditions [3], and has already been exploited in intra-operative applications, including scans of tissue specimens immediately after surgical resection [4].

Here we show that this technique also enables T staging esophageal tumors by producing images of resected human esophagi, asking two experienced radiologists to stage them blindly without knowledge of histopathological staging, and comparing the results with histopathology itself (taken as the ground truth). This resulted in a perfect match in all 14 tumor images considered in this study, all of which also visually correlated very well with their histopathology correspondent. We used a conservative scan time of 2 h, which would be compatible with the 6–7 h duration of a typical esophagectomy procedure; however, samples were immersed in ethanol overnight, which displaced some of the water, allowing for a better contrast within tissue layers. Currently this would create a barrier to intra-operative use, which would have to be overcome by developing a faster sample preparation procedure. As well as ensuring sample preservation for the following stages of histopathological analysis, the contrast enhancement resulting from immersion in ethanol was instrumental to the success of our study.

## 2. MATERIALS AND METHODS

All esophagectomy specimens were collected according to UK research guidelines. Ethical approval was granted by the London–Westminster Research Ethics Committee with University College London as the sponsor (ISRCTN registration 11347879, IRAS number 244284). Patients were identified by screening surgical lists, and written consent was obtained prior to surgery. Esophagectomy specimens collected from four patients were sufficient to provide a range of tumor stages, namely T1, T2, and T3 (see Table S1 in Supplement 1 for details). In all cases, samples were scanned with our XPCI system first, and then went through a standard histopathological workup, which included paraffin wax embedding and hematoxylin and eosin (H&E) staining.

In preparation for XPCI scanning, samples were sutured to rigid polylactic acid (PLA) scaffolds to ensure they were straight after fixation, and then underwent fixation in 10% formalin at UCL overnight. Following this they were dehydrated in graded ethanol for 4–30 h, according to Godkar’s rapid processing schedule [32], prior to transportation to Tring, where the XPCI scanner is located. During XPCI CT scans, samples were held in purpose-built, 3D-printed PLA tubes with 0.4 mm thick walls to minimize attenuation. The vertical length of the specimens often exceeded the 9 cm vertical field of view of our scanner.

**Fig. 1.**
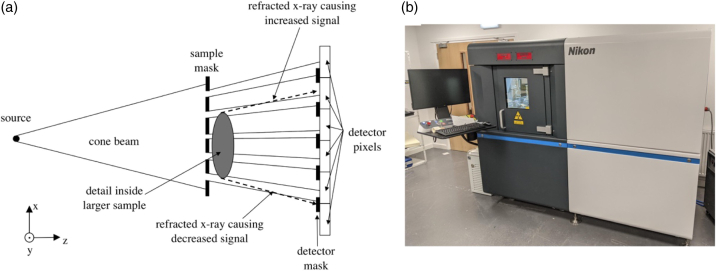
**(a)** Top-view conceptual schematic of edge-illumination x-ray phase contrast imaging and (b) photograph of the actual system.

The XPCI CT system was built by Nikon X-Tek Systems (Tring, United Kingdom), based on an earlier design developed at UCL [33]. It employs a rotating anode molybdenum source (Rigaku 007-HF Micro Max, Rigaku, Japan) with a focal spot size of approximately 70 µm, operated at 40 kVp with an applied current of 24 mA. An indirect conversion, CMOS-based flat panel detector (C9732DK-11, Hamamatsu, Japan) was used. The edge-illumination principle [31] was used to create phase sensitivity, employing two gold masks manufactured by Microworks GmbH (Karlsruhe, Germany) by electroplating a ∼120µm thick layer of gold on a 1 mm thick graphite substrate. The pre-sample mask was 9cm×9cm , with a period of 38 µm and an aperture size of 12 µm. The detector mask was 11.5cm×11.5cm in size, with a period of 48 µm and an aperture size of 20 µm. The sample mask, center of rotation of the sample stage, detector mask, and detector were placed at 65, 70, 80, and 83 cm from the source, respectively. Figure 1 shows a schematic of the setup alongside a photo of the scanner.

As can be seen in Fig. 1(a), the cone beam of x rays is split into multiple beamlets before it hits the sample. There is one beamlet for each (50 µm) detector pixel, so the original resolution (of approximately 100 µm [4], much coarser than histopathology) is not compromised and, in the absence of the sample, all pixels receive the same amount of x rays. When a sample is introduced, the phase changes it imposes on the x-ray beam causes some of the x rays to refract i.e., to deviate by a small angle. This effect is particularly strong at the interfaces of details inside a sample, as indicated by the two dashed lines in Fig. 1(a). The presence of a second mask in contact with the detector, which without the sample intercepts approximately 50% of the undeviated beamlets, means any such refraction effect is translated into a change in the intensity detected by the corresponding pixels. This change is accompanied by intensity reductions caused by attenuation; indeed, in a single image, the two effects are inherently mixed, and at least two images are required to separate them [34]. However, pioneering work by Paganin *et al*. [35] demonstrated that phase retrieval based on a single input image is possible if the sample is homogeneous, so that the phase and attenuation terms can be assumed to be proportional [i.e., γ(λ)=δ(x,y,z,λ)/μ(x,y,z,λ) is considered constant, where λ is the x-ray wavelength, δ is the unit decrement of the refractive index n=1−δ+iβ , and μ is the sample’s attenuation coefficient; see Supplement 1 for details); this is a reasonable approximation for esophageal samples. Diemoz *et al*. [36] adapted the Paganin approach to EI, a further development of which allowed phase contrast CT with laboratory sources to be performed in minutes [3].

It should be noted that, even though as a phase contrast method EI is achromatic [37], the acquisition of polychromatic CTs can still be affected by beam hardening artifacts, like for other XPCI methods [38]. In the results presented here, possibly owing to the Mo spectrum being dominated by the $k_{\alpha }$ and $k_{\beta }$ lines, cupping artifacts were not observed, and our focus is on the clear visualization of the muscle layers rather than a fully quantitative retrieval of δ . Another consequence of the polychromatic spectrum is the need to replace δ , μ , and γ with their “effective” counterparts $\delta_{eff}$ , $\mu_{eff}$ , and $\gamma_{eff}$ (see Supplement 1). The latter term needs to be set by the user; specifically, we selected the $\gamma_{eff}$ value that maximized the visibility of the muscle layers, and used the same value for all samples. This was $3.24\;*\;10^{-6}\;(\delta /\beta ∼10^{3})$ , which, according to the refractive index calculator of the University of Melbourne [39], matches that of soft tissue at an x-ray energy of approximately 20.7 keV, a reasonable assumption for the effective energy of our Molybdenum spectrum at 40 kVp.

All presented data correspond to a scan time of 2 h. Our protocol required us to perform 10-h scans to optimize prospective image quality as repeat scans were not possible (due to the need to return the specimens to the clinical pathway). Since the maximum exposure time of the detector was 0.6 s, 60,000 projections were taken to perform a 10-h scan. Datasets corresponding to 2-h scans were obtained by discarding 80% of the projections of a 10-h scan. Examples of images corresponding to even shorter scan times are provided in Supplement 1, Fig. S1.

Prior to scanning, an illumination curve is acquired without a sample present, by scanning the pre-sample mask while the detector mask is kept stationary. The pre-sample mask is then placed at one of the two positions corresponding to the maximum slope of this curve (on either side), which maximizes the system’s sensitivity to phase variations in the sample and remained stationary throughout the entire CT scan, thus allowing continuous sample rotation. Following phase retrieval of the individual projections, CT reconstruction is performed with standard filtered back-projections. Ring removal was applied to all scans by transferring the datasets to polar coordinates and applying median filtering. In most cases, additional ring removal was applied using Fourier filtering [40] (see Table S1 in Supplement 1 for details).

After XPCI scans, samples were returned to the clinical pathway and sent for histopathology.

The cancer stage was determined through histopathology as per the standard clinical practice described below, and through blind, independent radiological analysis by two radiologists with 21 (SAT) and 13 (MDC) years of experience in gastrointestinal cancer staging, based on the practice that is also described below. Radiologists were only presented with a single image for each considered case, corresponding to a scan time of 2 h as discussed above, processed with the same ring removal and phase retrieval algorithms with $\gamma_{eff}=3.24\;*\;10^{-6}$ .

In the gastro-intestinal tract, clinical staging (radiological and histological) is based on the extent that a tumor has invaded the tissues and spread. Carcinomas arise from the mucosal lining of the gut (specifically the epithelium) and from there invade into, and through, the different layers of the gut wall (mucosa, submucosa, muscularis propria, subserosa, and serosa). In the oesophagus the major landmark utilized for staging is the muscularis propria (main muscle coat). A tumor that has invaded the submucosa but has not invaded the muscularis propria is staged T1. If the tumor is seen extending into the muscularis propria it becomes T2 and if it extends beyond the muscularis propria T3. H&E staining is the traditional stain used by histopathologists to highlight structures in the tissues (basically haematoxylin stains cell nuclei and eosin cell cytoplasm). H&E delineates the different layers of the oesophagus wall allowing histopathologists to determine how deeply a tumor has invaded.

Radiologists segmented malignant tissue from background oesophageal tissue, on the basis that malignant tissue demonstrates altered x-ray characteristics relative to normal healthy oesophageal mucosa (primarily δ -related variations in tissue density, and evidence of distortion of the muscular layers). Mucosa, submucosa, muscularis, and serosa were then identified within normal, uninfiltrated oesophageal tissue to determine the depth of tumor invasion or number of mucosal layers crossed by each lesion. A corresponding T stage was then attributed for each lesion [41].

Following the above staging procedures, the radiologists (SAT and MDC) indicated the segmented tumor regions in the images, while the pathologist (MN) simply indicated with an arrow the region where the tumor invades into external tissue layers, as this is indicative of the tumor stage. While this led to some discrepancies, these regarded only a couple additional areas highlighted in some pathology slides, which, however, never affected the match between the staging ultimately assigned by the radiologists and the pathologist. These discrepancies are discussed on a case-by-case basis below and in Supplement 1.

For presentation in this paper and confirmation of the agreement between histopathology and XPCI results, XPCI CTs were matched to histopathology slices. This was done manually by reorienting the 3D dataset until a slice matching the morphological characteristics of the histopathology one could be identified. It should be noted that tissue often distorts during the paraffin embedding and cutting processes associated with histopathology (i.e., what appears as a flat slice in CT could be “bent” in the volume that is cut to produce the histopathology slices). On occasion, this can result in an imperfect match between the two slices, which can partly explain some of the discrepancies mentioned above. This notwithstanding, a good visual agreement is observed in the vast majority of the presented cases.

## 3. RESULTS

**Fig. 2.**
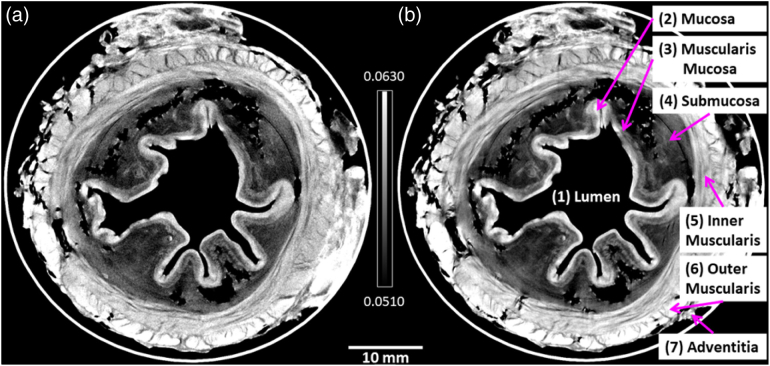
Example of cancer-free human esophagus with acquisition times of (a) 2 and (b) 10 h. Sections of the esophagus have been labeled in (b), with (1) the lumen, the space inside the esophagus; (2) mucosa, the membrane lining the interior of the esophagus; (3) muscularis mucosa, a thin layer of muscle separating the mucosa from the submucosa; (4) submucosa, a thin layer of connective tissue that supports the mucosa; (5) inner muscularis, the first of the two muscle layers with a circular arrangement; (6) outer muscularis, the second muscle layer with longitudinal alignment; and (7) adventitia, the outer layer of fibrous connective tissue. The color scale represents the attenuation coefficient $\mu_{eff}$ in ${mm}^{-1}$ [see Eq. (6) in Supplement 1], and the window width has been optimized to enhance the contrast of soft tissues. This has been applied in the same way to all presented images (which indeed all have a similar windowing); hence this is not repeated in the following captions.

Fourteen image datasets extracted from tumor-bearing specimens were blindly staged by the radiologists and, independently, through histopathology. A side-by-side comparison of CT versus histology slices is presented in all cases, either in the main body or as supplementary information. A cancer-free example is shown and labeled in Fig. 2, demonstrating that our method allowed good contrast to be generated through normal tissue. As can be seen, the contrast in and between layers is extremely high; the mucosa, sub-mucosa, and muscles layers are all well-defined, and the striations in the latter are apparent. Two- and 10-h scan durations are presented in Figs. 2(a) and (b) respectively. As the exposure time increases, the contrast-to-noise ratio increases as can be expected (roughly with the square root of exposure time); however, all key features are already detectable in the 2-h image of Fig. 2(a), with no additional features becoming visible thanks to the increased exposure time. As a result, all presented cancer-bearing CT slices correspond to a 2-h scan time, which has better compatibility with the requirements of intra-operative imaging. Examples of images corresponding to even shorter scan times are provided in Supplement 1, Fig. S1, for completeness.

In the following, we present example XPCI results side-by-side with their histopathology counterparts for tumor stages T1–T3; additional examples for all these stages are provided in Supplement 1.

**Fig. 3.**
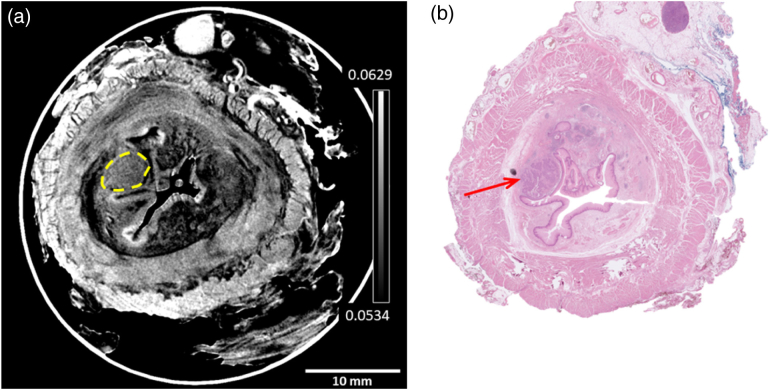
T1 cancer example, with (a) XPCI CT slide presented side-by-side with (b) its histopathology counterpart. In this and in the following images, tumor segmentation by the radiologists is shown in the CT image as a dashed yellow line, and an example of tissue invasion into the outer muscular layers (or lack thereof) identified by the pathologist is indicated with a red arrow in the histopathology slice. The color scale represents the attenuation coefficient $\mu_{eff}$ in ${mm}^{-1}$ (see caption of Fig. 2 for details).

The first example, presented in Fig. 3, is of a T1 cancer. The high contrast of all tissue layers in the XPCI CT slice of Fig. 3(a) is apparent, and there is a clear visual correlation with the features in the histopathology slice in Fig. 3(b), barring some deformation of the sample during the preparative cutting for histopathology, combined with the fact that, prior to histopathology workup, larger samples are sliced in half to ensure they fit on the histopathology sample stage. Muscle striations are clearly visualized in the XPCI CT image; a common feature of this and all other images presented below is that, in the tumor region [notable by the dark purple colors in Fig. 3(b)], this striation is lost, and nodes of the tumor become evident. The same region has been (blindly and independently) contoured in Fig. 3(a) by the two radiologists (MDC and SAT). In terms of staging, the red arrow in Fig. 3(b) shows that the tumor has just started to penetrate the supportive layer that divides the submucosa and muscle layers, indicative of a T1 stage cancer. Independent radiology review, blind to histopathology and based on the CT imaging alone, resulted in the same staging.

Three additional examples of T1 stage cancer are reported in Supplement 1, Fig. S2. In the first presented case [panels (a) and (b)], the pathologist has added a second arrow [see panel (b)] in a different part of the sample also affected by cancer, which is not visible in the CT image. This could be due to the tissue distortions caused by tissue embedding and cutting mentioned in the methods section, as the slightly different shape of the area in between two lobes of the lumen at around 7 o’clock seems to indicate. In the second example [panels (c) and (d)], while the tumor in the esophagus itself is staged as T1 by both radiologists and pathologists, a lymph node was also accidentally captured in the resection, and clear node involvement is detected both in the CT and in the histology. Nodal staging is beyond the scope of the present work, as it would require an accurate collection of an appropriate number of lymph nodes in all cases, but the potential value of this technique for nodal staging is demonstrated in this case. The third presented case [panels (e) and (f)] does not present any discrepancy. In summary, all these four examples (Fig. 3 plus Fig. S2) were staged as T1 by both pathologist and radiologists.

**Fig. 4.**
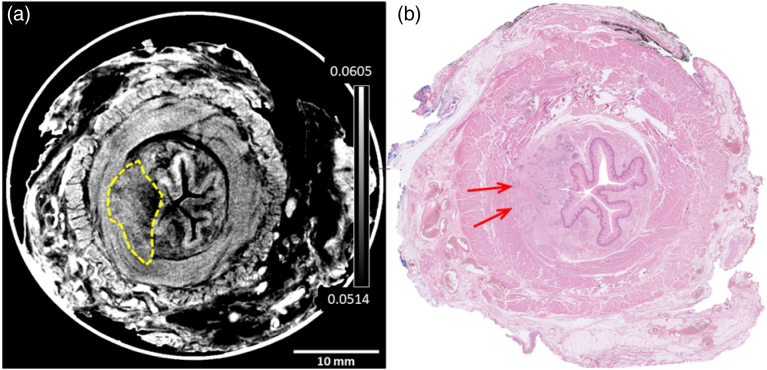
T2 cancer example, with (a) XPCI CT slide with lesion segmented by the radiologists presented side-by-side with (b) its histopathology counterpart, where two points where the tumor is just invading into the muscle layers have been highlighted with red arrows by the pathologist. The color scale represents the attenuation coefficient $\mu_{eff}$ in ${mm}^{-1}$ (see caption of Fig. 2 for details).

Figure 4 reports the same comparison for a stage T2 cancer.

Also in this case, the XPCI CT image (a) is presented alongside its histopathological counterpart (b), and the correspondence between features that can be observed in the two images can be appreciated. Both XPCI CT and histopathology slices make it evident that a slightly larger cancer than in Fig. 3 is present in this case, and the red arrows in Fig. 3(b) highlight the degree of its penetration into the underlying tissues. In particular, the tumor can be seen to reach into the muscle layers, which corresponds to a T2 stage. Three more examples are shown in Supplement 1, Fig. S3. While these present some minor discrepancies, individually discussed in Supplement 1, they were all staged as T2 both by the radiologists and the pathologist.

**Fig. 5.**
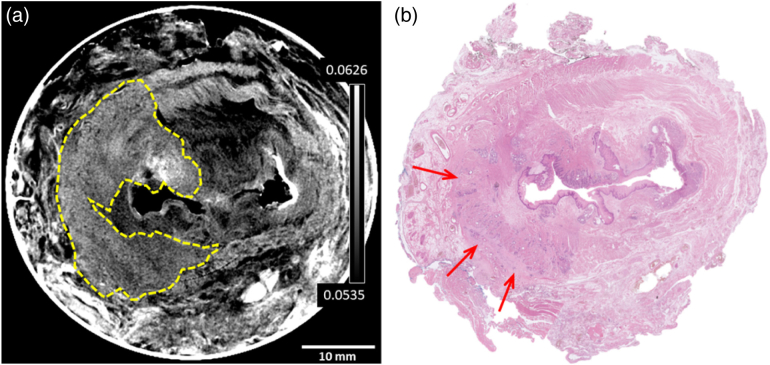
T3 cancer example, with (a) XPIC CT slide and segmented tumor presented side-by-side with (b) its histopathology counterpart. This is a much larger lesion than presented in previous cases, and the tumor invades into the adventitia at multiple points, three of which have been highlighted by the pathologist through red arrows. The color scale represents the attenuation coefficient $\mu_{eff}$ in ${mm}^{-1}$ (see caption of Fig. 2 for details).

Finally, the XPCI CT versus histopathology comparison for a stage T3 tumor is presented in Figs. 5(a) and 5(b), respectively. Here the disruption of the muscle layers is even more evident [see red arrows in Fig. 5(b)], and extends all the way into the adventitia, clearly indicative of T3 stage cancer. The differentiation between the different layers is completely disrupted across a good part of the sample, and this is evident both in the XPCI CT and in the histopathology slice.

Five additional examples of T3 cancers are provided in Supplement 1, Fig. S4, with no discrepancies between XPCI CT and histology slices other than a possibly smaller cancer area segmented by the radiologists in panel (g), which could again be due to sample misalignment and resulting worse match between CT and histopathology slices. All of these cases were staged as T3 by both radiologists and pathologists.

## 4. DISCUSSION AND CONCLUSIONS

The presented results show that the proposed technique acquires high-quality XPCI CT datasets that compare extremely well to the current histopathological gold standard, with the same features (or similar where deformation or slight mismatch has occurred) witnessed in both modalities. The contrast in and between esophageal layers is extremely high; the mucosa, sub-mucosa, and muscle layers all well-defined, and the striations in the latter are apparent. Based on the images in this study, it is possible to stage esophageal cancer using XPCI CT images alone: two experienced radiologists staged all XPCI CT images whilst blinded to histopathology, providing the same score in 14 out of 14 cases. It should be noted that histopathology provides features unattainable to the proposed x-ray method, such as the ability to resolve individual cells and to specifically highlight tissue types through targeted staining. In this sense, there is obviously a wide range of tasks that are attained through histopathology that are out of reach for the proposed x-ray method. The point we are making in this paper is that, for one specific task, i.e., T staging of esophageal tumors, the more limited information provided by our x-ray images may be sufficient.

All presented cases correspond to a scan time of 2 h. While this is potentially already compatible with intra-operative use in esophagectomy procedures, we expect that further system optimization would make it possible to bring the acquisition time down to 1 h or less. The ability to perform the imaging and staging intra-operatively is an important goal, because esophageal resections are associated with high morbidity and mortality. Achieving consistently clear resection margins is essential to improving curative outcomes. In particular, we note that the system used here was not specifically optimized for esophageal scans, but rather for the breast study presented in [4,5]; for example, the average x-ray energy could be increased through use of a different x-ray anode (e.g., W or Rh instead of Mo), which would increase the bremsstrahlung spectrum. It is worth noting that, in the breast study presented in [4], the initial 1-h-long scans were ultimately reduced to 10 min in the optimized prototype. Moreover, esophagectomy operations last approximately 6 h—much longer than wide local excisions in breast surgery—providing a longer time window for intra-operative imaging than the 10–15 min available in the latter case.

One obstacle that still needs to be removed in view of an intra-operative use of the technology is that, at the moment, specimens were immersed in ethanol overnight to displace some of the water, which has a very similar refractive index to muscle tissue. Establishing the minimum ethanol immersion time that guarantees sufficient image quality lies beyond the scope of the present work, and approaches to speed up ethanol absorption (e.g., through controlled temperature increases, which were proven to work for formalin [42]) or the use of different fluids (e.g., methanol was demonstrated to have a faster penetration rate than ethanol [43], with a possible alternative being xylene) should also be explored.

**Fig. 6.**
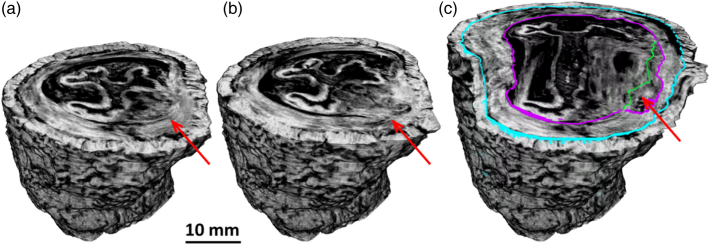
3D rendering of an esophagus section showing (a) slice where the cancer is contained to the submucosa and the muscle layer intact as shown by the red arrow, i.e., a T1 stage tumor; (b) slice further up where the muscle layer has been penetrated as shown by the red arrow, indicative of a stage T2 tumor; and (c) full volume. In (c), segmentation has been performed outlining the inner muscularis, with the pink and blue lines showing the inside and outside, respectively. The green line is an interpolation of the position of the inside of the muscularis from slices above and below where no tumor was present, meaning the area between green and pink highlighted by the red arrow in (c) is the T2 stage of the tumor.

The introduction of this technology into clinical practice could be beneficial for multiple reasons. First and foremost, the suitability for intraoperative use will enable real-time surgical response dependent on whether the entire tumor has been fully resected. Second, the ability to stage tumors during the operation would allow critical decisions that may impact directly on future management. Positive resection margins are associated with poor prognosis and may require adjuvant chemotherapy or tumor bed radiotherapy; the ability to determine the resection margins during the operation and carry out a more extensive resection, if necessary, could reduce the chance of cancer relapse and improve patient survival. Even if a complete resection is not possible because of anatomical constraints at the site of the involved resection margins, it would be possible to develop the technology to administer focused radiotherapy intra-operatively or new precision modalities post-operatively, such as proton beam therapy, which have become available at several centers worldwide.

Furthermore, the 3D images can be used as a significant aid to the pathologist, for example, in “right slice first time” approaches where features of interest are localized before the tissue is cut. It is also conceivable that, limited to areas where sufficient information has been safely obtained from the 3D XPCI dataset, the technology’s adoption could significantly reduce the burden and cost of histopathological workups.

The 3D images would also allow placement of histopathological information into a full 3D context, for example, in terms of the surrounding tumor environment. The method would provide pathologists and medical researchers with full, real 3D tumor images: at the moment, 3D visualization requires reassembling stacks of adjacent histopathological slices, which is time consuming and prone to inaccuracies because of artifacts associated with tissue slicing. A review of the advantages that advanced x-ray imaging could bring to histopathology is provided by Albers *et al*. [44].

Moreover, a tumor can present different stages at different positions along the esophagus, an example of which is provided in Fig. 6. In a case like this, sampling only certain slices for histopathological evaluation may lead to incorrect staging.

Clearly adoption of the proposed technology would require some degree of training for histopathologists and radiologists, or for them to work side by side; however, the relative similitude between XPCI and conventional CT slices (of which the former are basically a version with enhanced soft tissue contrast) should facilitate this process for radiologists. It is also conceivable that this process could be assisted and accelerated by the use of artificial intelligence-based approaches in the near future.

We conclude by listing some of the limitations of the current study. The main one is clearly the ethanol preparation of the specimens, already discussed in detail above, which at the moment takes too long to be compatible with intra-operative use. The ethanol associated water displacement also causes a shrinkage of both the healthy tissue and the tumor, which could have an influence on our study. Some reassurance is provided by the fact that, as explained in Section 2, T staging is based on the assessment of whether the tumor has infiltrated through specific tissue layers. Being able to correctly visualize each layer enables determining whether they are intact or affected by the tumor, which should not change when both the tumor and surrounding tissue shrink.

Although the staging matched in 14 out of 14 of the cases, some discrepancies were observed between histopathology and XPCI images; while some of these are probably due to a mismatch between the considered slices, this aspect should be given further consideration before the method could be used clinically.

Another limitation is the small number of patients that were involved in the study, and consequently the small number of images that were ultimately used (see Table S1 in Supplement 1). We note, however, that this work had no ambitions to be a clinical study, but rather to provide the proof-of-concept that esophageal tumors could be staged with phase contrast x rays. All specimens came from esophagectomies, so the pathologist and the radiologists knew they were expecting a tumor, and were only asked to stage it–again to fulfill the “proof-of-concept” aim stated above. All patient data were anonymized; due to the extremely small cohort, data such as age or gender could be considered identifiers, which is why they are not reported in Table S1.

## Supporting information

10.6084/m9.figshare.25563903 Supplement 1 Additional document with more data. https://doi.org/10.6084/m9.figshare.25563903

## Data Availability

The datasets generated and/or analyzed during the current study are available from the corresponding author on reasonable request.

## Supplemental document

See Supplement 1 for supporting content.
