# Supplementary material for: T staging esophageal tumors with x rays
Source: Optica. 2024 Apr 19;11(4):569–76. doi: 10.1364/OPTICA.501948 (PMC11239146; doi:10.1364/OPTICA.501948)
Supplement: Supplementary file 1 [file optica-11-4-569-s001.pdf]

Supplemental Document

OPTICA

# T staging esophageal tumors with x rays: supplement

T. Partridge,[1] P. Wolfson,[1,2] J. Jiang,[1,3] L. Massimi,[1] A. Astolfo,[1,4] N. Djurabekova,[5] S. Savvidis,[1] C. J. Maughan Jones,[1] C. K. Hagen,[1] E. Millard,[4] W. Shorrock,[4] R. M. Waltham,[4] I. G. Haig,[4] D. Bate,[1,4] K. M. A. Ho,[2] H. Mc Bain,[2] A. Wilson,[2] A. Hogan,[2] H. Delaney,[6] A. Liyadipita,[6] A. P. Levine,[6] K. Dawas,[7] B. Mohammadi,[7] Y. A. Qureshi,[7] M. D. Chouhan,[8,9,10] S. A. Taylor,[8] M. Mughal,[7] P. R. T. Munro,[1] M. Endrizzi,[1] M. Novelli,[11] L. B. Lovat,[2] and A. Olivo[1,*]

[1] *Department of Medical Physics and Biomedical Engineering, University College London, London WC1E 6BT, UK*
[2] *Division of Surgery and Interventional Science, UCL, London WC1E 6BT, UK*
[3] *Current address: Advanced Photon Source, Argonne National Laboratory, Lemont, Illinois 60439, USA*
[4] *Nikon X-Tek Systems Ltd., Tring, Herts HP23 4JX, UK*
[5] *Department of Computer Science, UCL, London WC1E 6BT, UK*
[6] *Department of Histopathology, UCL, London WC1E 6BT, UK*
[7] *Department of Upper Gastro-Intestinal Surgery, UCLH, London NW1 2BU, UK*
[8] *Center for Medical Imaging, Division of Medicine, UCL, London WC1E 6BT, UK*
[9] *Princess Alexandra Hospital Medical Imaging Department, Brisbane, Queensland, Australia*
[10] *University of Queensland Medical School, Saint Lucia, Queensland, Australia*
[11] *Research Department of Pathology, Cancer Institute, UCLH, London NW1 2BU, UK*
[*] a.olivo@ucl.ac.uk

This supplement published with Optica Publishing Group on 19 April 2024 by The Authors under the terms of the Creative Commons Attribution 4.0 License in the format provided by the authors and unedited. Further distribution of this work must maintain attribution to the author(s) and the published article's title, journal citation, and DOI.

Supplement DOI: https://doi.org/10.6084/m9.figshare.25563903

Parent Article DOI: https://doi.org/10.1364/OPTICA.501948

## Supplementary Information for: T staging esophageal tumors with x-rays

T. Partridge[1], P. Wolfson[1,2], J. Jiang[1*], L. Massimi[1], A. Astolfo[1,3], N. Djurabekova[4], S. Savvidis[1], C.J. Maughan Jones[1], C.K. Hagen[1], E. Millard[3], W. Shorrock[3], R.M. Waltham[3], I.G. Haig[3], D. Bate[3,1], K.M.A. Ho[2], H. Mc Bain[2], A. Wilson[2], A. Hogan[2], H. Delaney[5], A. Liyadipita[5], A.P. Levine[5], K. Dawas[6], B. Mohammadi[6], Y.A. Qureshi[6], M.D. Chouhan[7,8,9], S.A. Taylor[7], M. Mughal[6], P.R.T. Munro[1], M. Endrizzi[1], M. Novelli[10], L.B. Lovat[2], A. Olivo[1**]

[1]Department of Medical Physics and Biomedical Engineering, UCL, London, WC1E 6BT, UK
[2]Division of Surgery and Interventional Science, UCL, London, WC1E 6BT, UK
[3]Nikon X-Tek Systems Ltd, Tring, Herts, HP23 4JX, UK
[4]Department of Computer Science, UCL, London, WC1E 6BT, UK
[5]Department of Histopathology, UCL, London, WC1E 6BT, UK
[6]Department of Upper Gastro-Intestinal Surgery, UCLH, London NW1 2BU, UK
[7]Center for Medical Imaging, Division of Medicine, UCL, London, WC1E 6BT, UK
[8]Princess Alexandra Hospital Medical Imaging Department, Brisbane, Queensland, Australia
[9]University of Queensland Medical School, Saint Lucia, Queensland, Australia
[10]Research Department of Pathology, Cancer Institute, UCLH, London NW1 2BU, UK
[*]current address: Advanced Photon Source, Argonne National Laboratory, Lemont, IL 60439
[**]corresponding author (a.olivo@ucl.ac.uk)

**Table S1** Table matching the figures presented in the article with the esophageal specimens they were taken from, tumor stage at that specific position along the esophagus (corresponding to the presented CT slice), and ring removal procedure that was applied. All samples come from esophagectomies, meaning all patients were diagnosed with esophageal cancer; however, specimens typically also contained cancer-free regions due to lesions being localized. Similarly, different parts of the same specimen may contain lesions at different stages (see Fig. 6 in the main article and related discussion). The radiologists were expecting to find a tumor, and their task was to stage it. Four specimens were sufficient to provide a range of tumor stages, namely T1, T2, and T3; this was considered sufficient for a technical study aimed at obtaining proof-of-concept that esophageal tumors could be staged by means of X-ray images alone (as opposed to a clinical study). Instances of T4 cancer were not observed, which is not surprising as stage T4 patients are typically considered unsuitable for esophagectomy.

| Figure | Patient | stage | Ring Removal |
|---|---|---|---|
| 2 | A | N.A. | Polar |
| 3 | B | T1 | Fourier & Polar |
| 4 | C | T2 | Fourier & Polar |
| 5 | C | T3 | Fourier & Polar |
| S2(a) | D | T1 | Fourier & Polar |
| S2(c) | B | T1 | Fourier & Polar |
| S2(e) | C | T1 | Fourier & Polar |
| S3(a) | D | T2 | Fourier & Polar |
| S3(c) | B | T2 | Fourier & Polar |
| S3(e) | B | T2 | Fourier & Polar |
| S4(a) | B | T3 | Fourier & Polar |
| S4(c) | C | T3 | Fourier & Polar |
| S4(e) | C | T3 | Fourier & Polar |
| S4(g) | E | T3 | Fourier & Polar |
| S4(i) | B | T3 | Fourier & Polar |

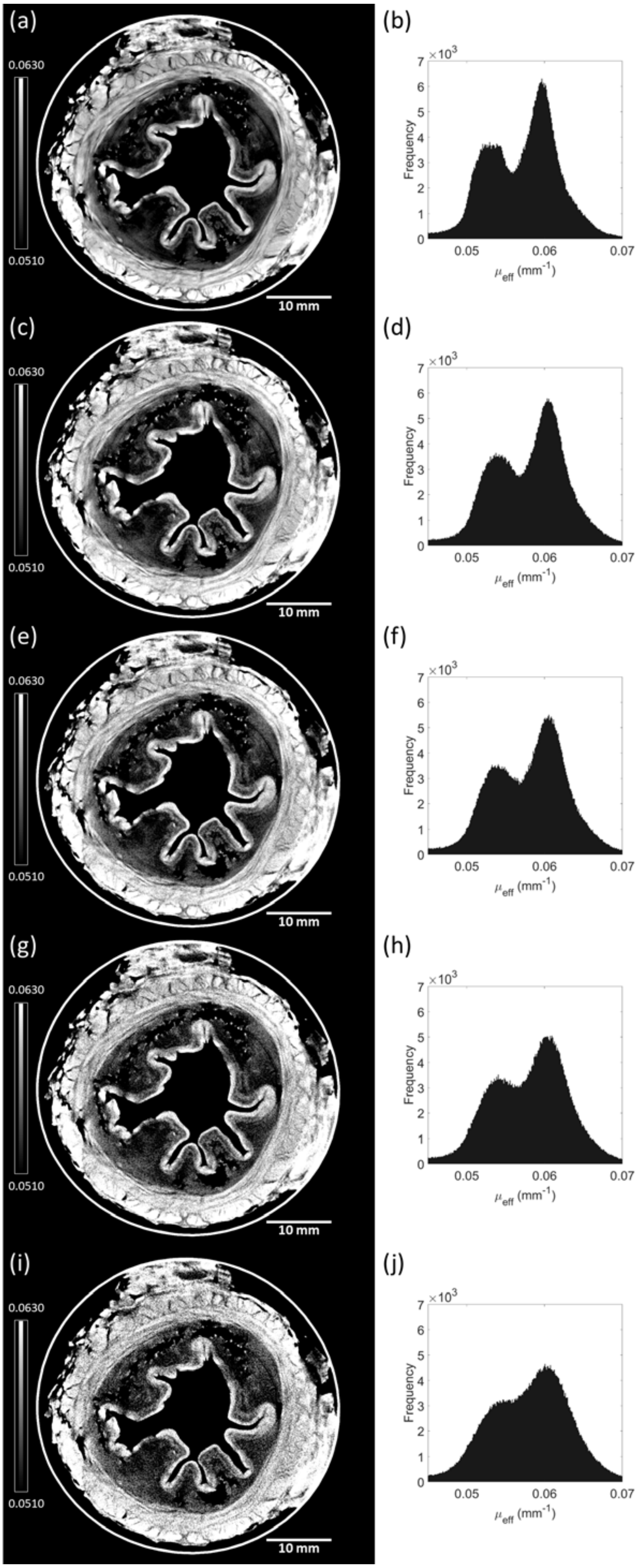

(a)
0.0630
0.0510
10 mm
(b)
×10³
Frequency
0
1
2
3
4
5
6
7
0.05
0.06
0.07
μeff (mm⁻¹)
(c)
0.0630
0.0510
10 mm
(d)
×10³
Frequency
0.05
0.06
0.07
μeff (mm⁻¹)
(e)
0.0630
0.0510
10 mm
(f)
×10³
Frequency
0.05
0.06
0.07
μeff (mm⁻¹)
(g)
0.0630
0.0510
10 mm
(h)
×10³
Frequency
0.05
0.06
0.07
μeff (mm⁻¹)
(i)
0.0630
0.0510
10 mm
(j)
×10³
Frequency
0.05
0.06
0.07
μeff (mm⁻¹)

**Fig. S1:** Images corresponding to further reductions in scan time compared to Fig 2 in the main article, obtained by discarding an increasing number of projections, alongside their corresponding grey level histograms. Images in panels (a), (c), (e), (g) and (i) correspond to 10 hours, 2 hours (same as in Fig 2, repeated here to allow a direct comparison with the followingimages), 1 hour, 30 minutes and 15 minutes, respectively. Each image is accompanied by a histogram representing the corresponding distribution of pixel content ($\mu_{eff}$ in $mm^{-1}$, see below; panels (b), (d), (f), (h) and (j) for images in panels (a), (c), (e), (g) and (i), respectively). As can be seen, grey levels are not significantly affected by the angular down-sampling in the images; the grey level distribution in the histograms becomes increasingly smoothed out with reduced scan times, indicative of the reduced contrast-to-noise ratio. Although reasonable quality images are still obtained with datasets corresponding to scan times as short as 30 and possibly even 15 minutes, it should be noted that e.g. the 30 minute ones were obtained with 3,000 projections, which is already slightly below the recommended number of CT projections when a detector with 2,400 pixels is used. A correct optimization of scans performed in shorter times should entail collecting more projections with a shorter acquisition time per projection, which was made impossible by our protocol imposing us to perform a single, long scan for each sample (see main article). The color scale represents the attenuation coefficient $\mu_{eff}$ in $mm^{-1}$ (see equation (6) below), and the window width has been optimized to enhance the contrast of soft tissues. This has been applied in the same way to all presented images (which indeed all have a similar windowing), hence this is not repeated in the following captions.

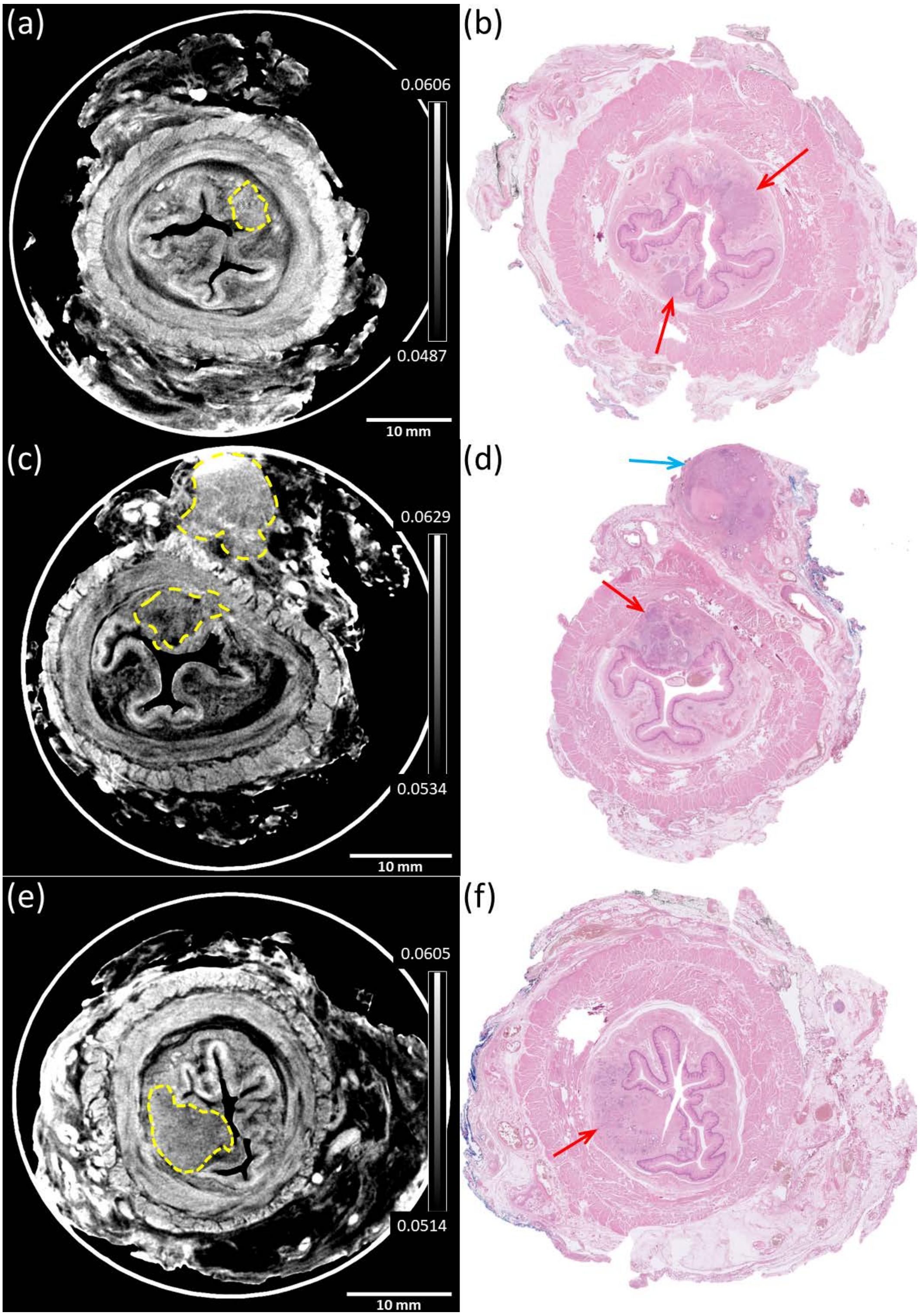


**Fig. S2:** Three more examples of stage T1 esophageal tumors, with XPCI CT slices (a, c, e) juxtaposed to their corresponding histopathological slices (b, d, f). In all cases, the tumor has been segmented by the radiologist in the CT slices, while the pathologist has put a red arrow in the histology slices indicating the point where the tumor has just started to invade the supportive layer that divides the submucosa and muscle layers, indicative of a T1 stage cancer. In panel (b), an additional arrow (at roughly 7 o'clock) highlights a second (T1) tumor formation which is not visible in the CT slice in (a), either because it was not detected, or

because tissue distortions associating with the embedding and cutting required by histology meant the lesion ended up outside the considered CT slide. Since similar lesions are regularly detected in all considered samples (with the lesion at 2 o'clock in the same sample providing a good example), we would be inclined to consider the latter explanation as more likely. In panel (c), a peripheral lymph node is highlighted by the radiologists; this was indeed an affected lymph node, as is clearly visible (and highlighted with a blue arrow by the pathologist) in the histology counterpart in panel (d). While on the one hand this hints at the possible inadequacy of T staging alone, it also suggests the possibility of using the technology for N staging, so long as a sufficient number of lymph nodes are collected and scanned – however, this laid beyond the scope (and ethics protocol) of the current work. The color scale represents the attenuation coefficient $\mu_{eff}$ in mm$^{-1}$ (see caption of Fig S1 for details).

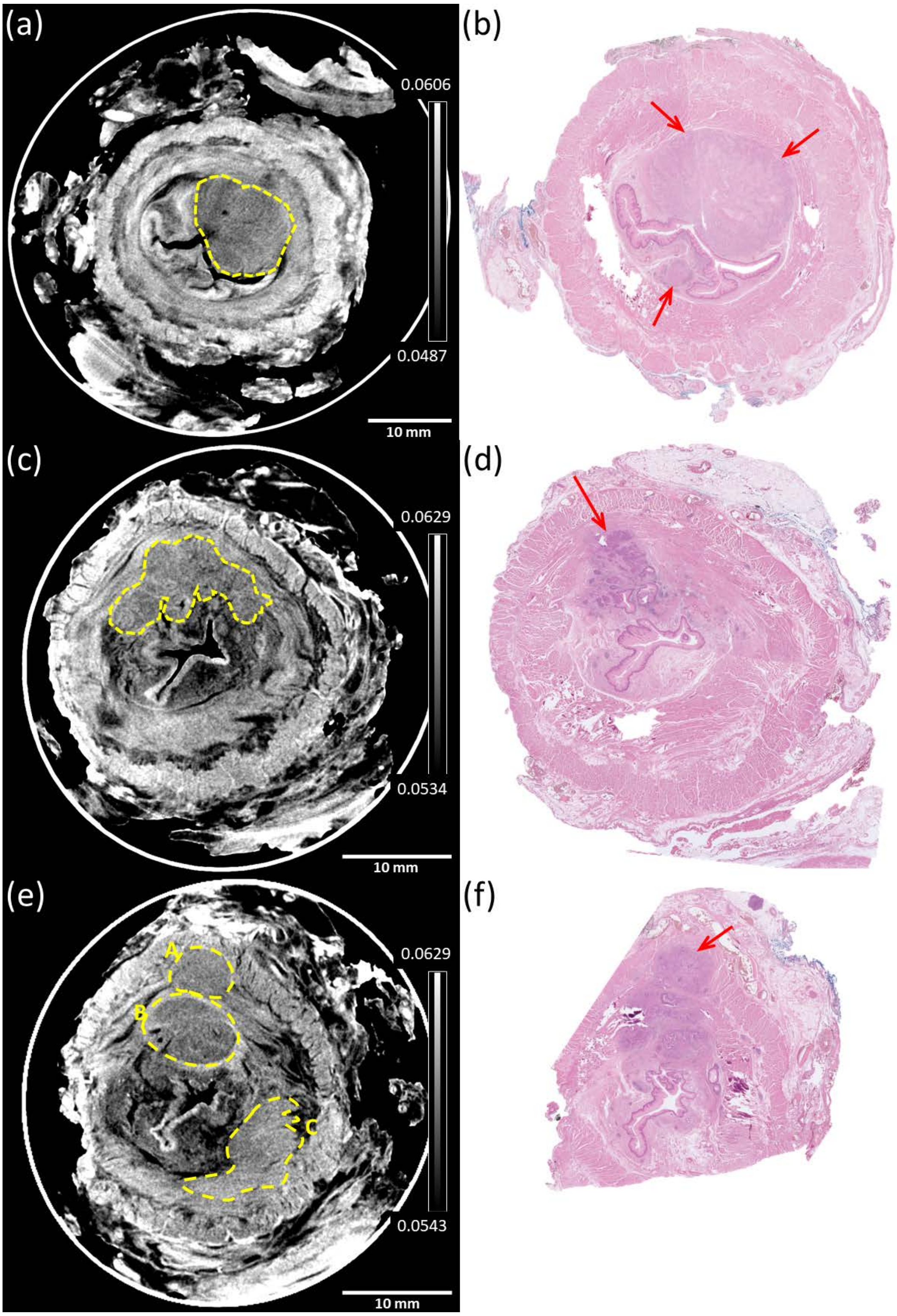


**Fig. S3:** Additional examples of stage T2 esophageal tumors, with XPCI CT slices (a, c, e) next to the corresponding histopathological slices (b, d, f). The segmented areas in (a) and (c) match the corresponding areas in (b) and (d), in which arrows drawn by the radiologist point at areas where the tumor is invading into the muscle layers, indicative of T2 cancer. The pathologist highlights an additional area in (b), corresponding to a lesion that is not

visualized in (a). This is a similar situation to that observed in Fig. S1 (b), however it should be noted that the match between CT and histology slice is worse in this case, as it was more difficult to select exactly the same area from the x-ray dataset (e.g. see the differences in the lumen's shape, especially around 9 o'clock, and the significantly reduced area in between the two lumen "branches"). In panel (e), the radiologists segmented three different regions, labelled as A, B and C. Region A corresponds to that highlighted by the pathologist with an arrow, as it is the one where the tumor invades into the muscle layer. The lesion of region B is clearly visible in panel (f), although it had not been highlighted by the pathologist since it does not invade into the outer layers. Finally lesion C is mostly missed as half of the histopathology slice was missing in this case. The color scale represents the attenuation coefficient $\mu_{eff}$ in $mm^{-1}$ (see caption of Fig S1 for details).

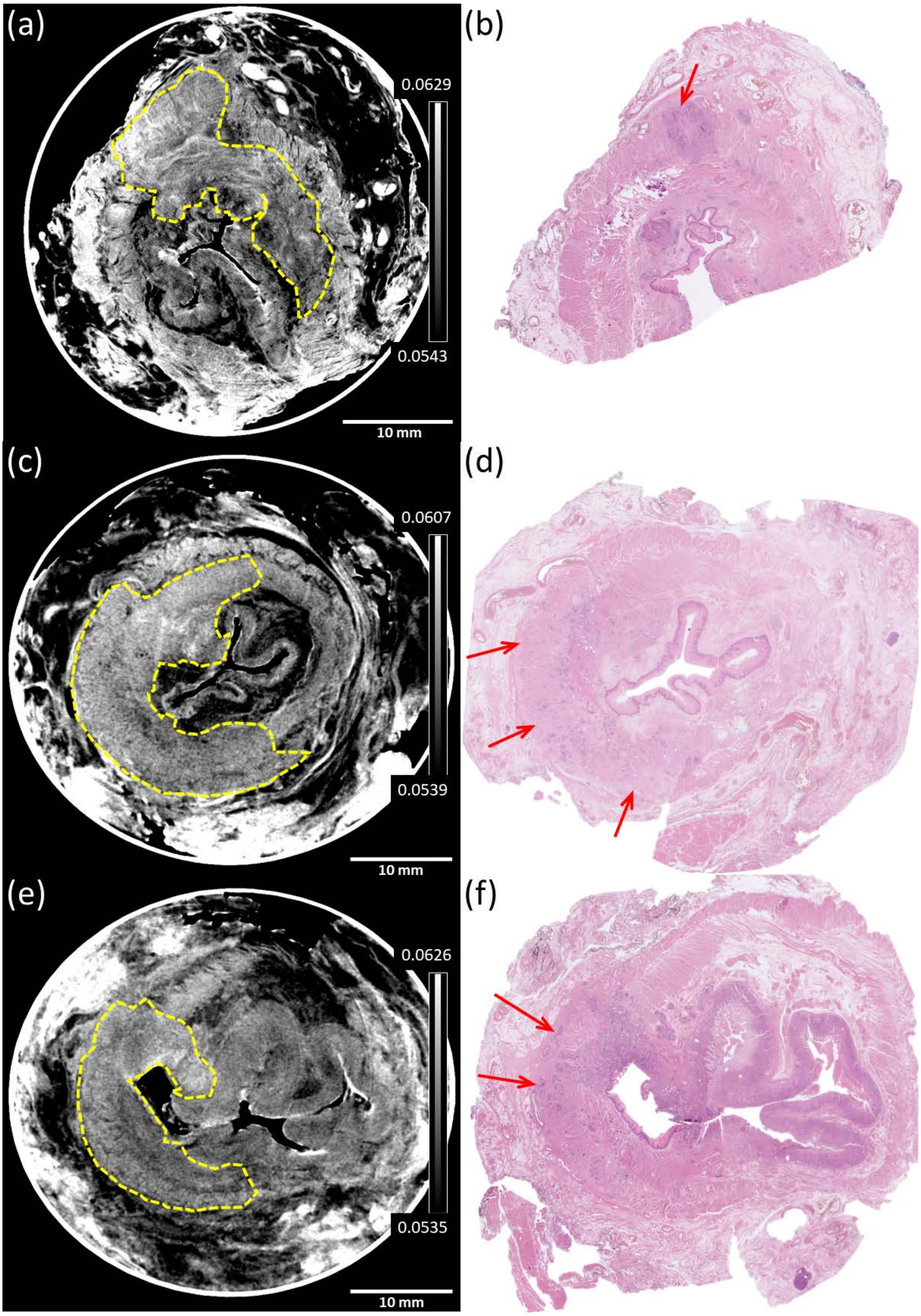
(a)
(b)
0.0629
0.0543
10 mm
(c)
(d)
0.0607
0.0539
10 mm
(e)
(f)
0.0626
0.0535
10 mm

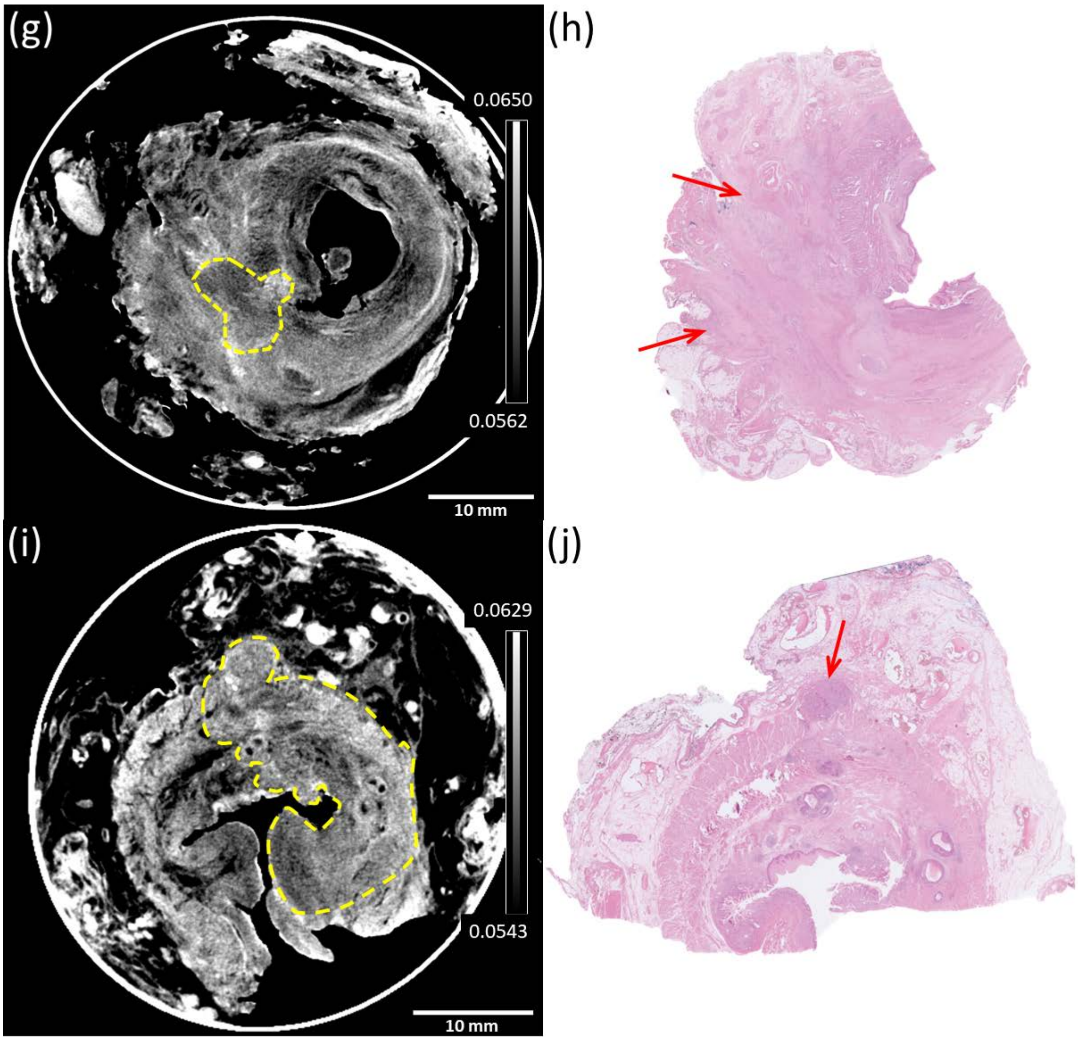


**Fig. S4:** Additional examples of stage T3 esophageal tumors, with XPCI CT slices (a, c, e, g, i) next to the corresponding histopathological slices (b, d, f, h, j). In all these examples, the tumor is very extensive and significantly disrupt the submucosa and both muscle layers invading into the adventitia (see red arrows by the pathologist in panels (b, d, f, h, j)), corresponding to T3 stage cancer; indeed, all these cases were stages ad T3 by both radiologists and pathologist. As mentioned in the main manuscript, specimens were too large to fit into a single histological slice, and as a result the slices shown in this article are obtained by pasting together two separate "half" slices. Because of this, half a slice is missing from panels (b), (h) and (j) – however the available half is sufficient to observe tumor invasion into the various layers. All cases are straight matches, with the pathologist using one or more arrows depending on the size of the "front" of the tumor invading into the adventitia. Panel (g) shows a case where the radiologists have segmented a smaller area, which again could be due to the worse match between CT and histological slices. Also in this case, however, they have staged the tumor as T3, in (blind) agreement with the pathologist. The color scale represents the attenuation coefficient $\mu_{eff}$ in $mm^{-1}$ (see caption of Fig S1 for details).

**Brief description of the underpinning mathematical model:**

If one introduces the illumination curve $C(x, \lambda)$, obtained by scanning the pre-sample mask along the direction of phase sensitivity (i.e. $x$, see Fig 1(a) in the main article, with $\lambda$ the x-ray wavelength) and recording the detected x-ray intensity, the intensity detected in the presence of a sample on position $\bar{x}$ of the illumination curve can be written as [1]:

$$I(x,y,\lambda) = \Big\{\exp[-p(x,y,\lambda)] - \exp[-p(x,y,\lambda)]\frac{C'(\bar{x},\lambda)}{C(\bar{x},\lambda)}\frac{\bar{z}}{k}\nabla_x\Phi(x,y,\lambda) - \frac{\bar{z}}{k}\nabla_y\big[\exp[-p(x,y,\lambda)]\nabla_y\Phi(x,y,\lambda)\big]\Big\} * L(y) \quad (1)$$

With $y$ the coordinate along the mask apertures (entering the plane of the drawing in Fig 1(a)), $C'$ the first derivative of $C$, $\bar{z}$ the sample to detector mask distance, $k$ the wave number, $\nabla_x$ and $\nabla_y$ the gradient operators along $x$ and $y$ respectively. $L(y)$ is the detector's Line Spread Function (LSF) along $y$, and * the (one-dimensional) convolution operator; $\exp[-p(x,y,\lambda)]$ expresses the local sample transmission $T(x,y,\lambda)$, with $p(x,y,\lambda)=\int \mu(x,y,z,\lambda)dz$ where $z$ is the x-ray propagation direction, $\mu$ the sample's attenuation coefficient and the integral extends over the depth of the sample along $z$. Finally $\Phi(x,y,\lambda)$ is the sample's induced phase shift:

$$\Phi(x,y,\lambda) = -\mathrm{k}\int \delta(x,y,z,\lambda)dz \quad (2)$$

with $\delta$ the unit decrement of the refractive index $n = 1 - \delta + i\beta$, and again with the integral extending over the depth of the sample along $z$. For a homogeneous sample, one can assume the ratio $\gamma(\lambda) = \delta(x,y,z,\lambda)/\mu(x,y,z,\lambda)$ to be constant over the field of view; under this assumption, $\Phi(x,y,\lambda)$ can be written as $-\mathrm{k}\int \gamma\mu(x,y,z,\lambda)dz = -k\gamma(\lambda)p(x,y,\lambda)$. By omitting the dependence on spatial coordinates for simplicity, (1) becomes:

$$I(\lambda) = \Big\{\exp[-\mathrm{p}(\lambda)] + \exp[-\mathrm{p}(\lambda)]\frac{C'(\lambda)\gamma(\lambda)\bar{z}}{C(\lambda)}\nabla_x\mathrm{p}(\lambda) + \gamma(\lambda)\bar{z}\nabla_y\big[\exp[-p(\lambda)]\nabla_y p(\lambda)\big]\Big\} * L(y) \quad (3)$$

By observing that $\exp[-p(\lambda)]\,\nabla_{x,y}p(\lambda) = -\nabla_{x,y}[\exp[-p(\lambda)]]$, (3) becomes:

$$I(\lambda) = \Big\{\exp[-\mathrm{p}(\lambda)] - \frac{C'(\lambda)\gamma(\lambda)\bar{z}}{C(\lambda)}\nabla_x[\exp[-\mathrm{p}(\lambda)]] - \gamma(\lambda)\bar{z}\nabla_y^2[\exp[-p(\lambda)]]\Big\} * L(y) \quad (4)$$

We can now take the Fourier transform ($\mathcal{F}$) of both sides of eq (4), while considering that the unidirectional line spread function $L(y)$ becomes the modulation transfer function along the corresponding spatial frequency $M(f_y)$, that the convolution becomes a product, and that the Fourier transform of $d^nF(x)/dx^n$ is equal to $(2\pi i f_x)^n F(x)$ (with $f_x$, $f_y$ the frequencies associated with spatial coordinates $x,y$):

$$\mathcal{F}[I(\lambda)] = M(f_y)[1 - \frac{2\pi i f_x C'(\lambda)\gamma(\lambda)\bar{z}}{C(\lambda)} + 4\pi^2 f_y^2\gamma(\lambda)\bar{z}]\mathcal{F}[\exp[-p(\lambda)]] \quad (5)$$

Eq. (5) is only valid for monochromatic radiation. To apply it to a polychromatic source, one needs to 1) consider that the EI method is achromatic, a fully valid assumption if transmission through the masks can be neglected [2], which means *C* (and therefore *C'*) do not depend on $\lambda$, and 2) by replacing $\mu$ and $\gamma$ with their "effective" counterparts $\mu_{eff}$ and $\gamma_{eff}$, obtained as the spectrally weighted sum of all monochromatic components [1,3]. This allows accessing the line integrals required for CT reconstruction by taking the logarithm of the inverse Fourier transform of the remaining terms:

$$p_{eff}=\int \mu_{eff}(x,y,z)dz=-\ln\left[\mathcal{F}^{-1}\left\{\frac{\mathcal{F}[I]}{M(f_y)[1-\frac{2\pi i f_x C' \gamma_{eff}\bar{z}}{C}+4\pi^2 f_y^2 \Upsilon_{eff}\bar{z}]}\right\}\right] \tag{6}$$

Once $\mu_{eff}$ is volumetrically reconstructed, then of course $\delta_{eff} = \gamma_{eff}\mu_{eff}$.

## References


1. P. Diemoz, C.K. Hagen, M. Endrizzi, M. Minuti, R. Bellazzini, L. Urbani, P. De Coppi, and A. Olivo, "Single-Shot X-Ray Phase-Contrast Computed Tomography with Nonmicrofocal Laboratory Sources," Phys. Rev. Appl. **7**(4), 044029 (2017).
2. M. Endrizzi, F.A. Vittoria, G. Kallon, D. Basta, P.C. Diemoz, A. Vincenzi, P. Delogu, R. Bellazzini and A. Olivo, "Achromatic approach to phase-based multi-modal imaging with conventional x-ray sources," Opt. Exp. **23**(12), 16473-16480 (2015).
3. A. Olivo and R. Speller, "Experimental validation of a simple model capable of predicting the phase contrast imaging capabilities of any x-ray imaging system," Phys. Med. Biol. **51**(12), 3015-3030 (2006).
